# A Parallel Cellular Automata Lattice Boltzmann Method for Convection-Driven Solidification

**DOI:** 10.1007/s11837-018-3195-3

**Published:** 2018-11-12

**Authors:** Andrew Kao, Ivars Krastins, Matthaios Alexandrakis, Natalia Shevchenko, Sven Eckert, Koulis Pericleous

**Affiliations:** 1 Centre for Numerical Modelling and Process Analysis, University of Greenwich, Old Royal Naval College, Park Row, London, SE109LS UK; 2 Institute of Fluid Dynamics, Helmholtz-Zentrum Dresden-Rossendorf, Bautzner Landstrasse 400, 01328 Dresden, Germany

## Abstract

This article presents a novel coupling of numerical techniques that enable three-dimensional convection-driven microstructure simulations to be conducted on practical time scales appropriate for small-size components or experiments. On the microstructure side, the cellular automata method is efficient for relatively large-scale simulations, while the lattice Boltzmann method provides one of the fastest transient computational fluid dynamics solvers. Both of these methods have been parallelized and coupled in a single code, allowing resolution of large-scale convection-driven solidification problems. The numerical model is validated against benchmark cases, extended to capture solute plumes in directional solidification and finally used to model alloy solidification of an entire differentially heated cavity capturing both microstructural and meso-/macroscale phenomena.

## Introduction

With increasing parallel computational power, microstructure modeling now has the capability to reach meso- or even macroscale dimensions. In practice, this means a single simulation can simultaneously examine both micro- and macroscale phenomena and their various interactions. In many cases, this removes the need for boundary condition approximations as the computational domain can encompass entire components or experiments. To bridge the gap in scales, ranging from dendritic *O*(0.1 mm–1 mm) to component *O*(1–10 cm), there are two key approaches. The first is the operating length scale of the numerical methods, which to achieve this goal should be the largest for capturing microscopic phenomena. The second is parallelization, which provided the method scales well with increasing processers allows for increased domain sizes. On a practical level though, simulating a very large number of computational cells is restricted to large high-performance computing clusters. The aim here is to combine these two approaches to allow timely simulations of small-scale components or experiments.

The phase field method is arguably the most accurate microstructural approach. Several investigators have parallelized this and modeled cases using billions of calculation cells. Takaki et al.^1^ used 64 billion cells (4096^3^) to simulate solidification without convection in a $3.072 \times 3.078 \times 3.072$-mm domain. Shimokawabe et al.^2^ developed a hybrid-parallelized phase field method that uses the Message Passing Interface (MPI) and CUDA™. By using 16,000 central processing unit (CPU) cores and 4000 graphical processing units (GPU), they achieved a computational domain of 277 billion cells with a physical size of $3.072 \times 4.875 \times 7.8\,{\text{mm}}^{3}$. Sakane et al.^3^ developed a GPU parallelized lattice Boltzmann phase field method and simulated 7 billion mesh points, with a real size of $61.5 \times 61.5 \times 123\;\mu {\text{m}}^{3}$, using 512 GPUs. Choudhury et al.^4^ compared a parallel cellular automata method with a parallel phase field method, showing that the cellular automata method was faster in comparable 3D free undercooled growth. Similar success has been achieved with a 3D parallel lattice Boltzmann cellular automata method^5^ simulating solute-driven multi-dendritic growth. By using 6400 cores they simulated a domain of 36 billion cells with a volume of $1\;{\text{mm}}^{3}$.

Sun et al.^6^ proposed a cellular automata Lattice boltzmann (CA-LB) model to simulate dendritic solidification in 2D. They used the lattice Boltzmann method (LBM) to describe the mass and momentum transport in an undercooled crystal growth. Later they included the heat transfer to simulate single- and multi-dendritic growth of binary alloys with melt convection^7^ and recently investigated the effect of melt convection on multi-dendritic growth without considering temperature differences in the simulation domain.^8^ The mesh sizes they used are all sub-micron ranging from 0.125 µm to 1 µm.

Yin et al.^9^ also used the CA-LB method (CALBM) to simulate solidification at the microscale in 2D. They compared the efficiency of the CA-LB model against the finite element-CA model and concluded that the CALBM is much more efficient when fluid flow is being considered.

Sun et al. continued working on the CALBM expanding it to 3D to model directional solidification of binary alloys. They investigated tip-splitting of the dendrite tips caused by high solidification rates^10^ and studied the bubble formation in dendritic growth.^11^ In their studies they employed the popular D3Q19 lattice to describe the mass and momentum transport; however, their spatial step and time interval were chosen as 0.5 µm and 0.1 µs, which is typical for phase field method.

The main focus of parallelization efforts has been the phase field method, which remains a strictly microscopic method even after massive parallelization. Parallel cellular automata methods have been developed but test only a small computational domain and investigate idealized cases. In this work, the goal is to develop a method that is comparable to those in the literature and apply it to capture an entire experiment.

For solidification, the cellular automata method (CAM) adopted is based on the open source code µMatIC.^12^–^15^ This method, while sacrificing some accuracy compared with phase field methods, has practical uses as it can produce realistic results on cell sizes an order of magnitude larger. Given equivalent large-scale computational resources, then macroscopic domain sizes could be simulated successfully.

To achieve this goal, the core solidification solver of µMatIC was extracted and parallelized.^16^ The CAM uses a decentered octahedral method to simulate dendritic solidification across crystallographic directions of equiaxed metals and alloys, originally developed using finite elements by Gandin and Rappaz.^17^,^18^ Wang et al.^14^ modified the decentered octahedral method in the Imperial College µMatIC code to couple the CAM with a finite difference (FD) solver to account for solute diffusion. Yuan and Lee^19^,^20^ further coupled the modified CA-FD model with a fluid flow solver to account for forced and natural convection and studied the initiation and formation of freckles in the microstructure of directionally solidified Pb–Sn alloys,^21^ while Karagadde et al.^22^ investigated the formation of freckles in the microstructure of directionally solidified Ga-25wt.%In alloy.

Resolving fluid flow in dendritic solidification is computationally expensive, requiring handling of evolving intricate geometries in the flow space. In this work, the LBM is used to model the fluid flow. The LBM, as described by kinetic theory, is inherently transient and is well suited for meso- and microscale problems. The method has become increasingly attractive because of its simplicity, efficiency, versatility and because it lends itself to massive parallelization. With recent advances in parallel computing power, the LBM can be faster than conventional computational fluid dynamics (CFD) methods, especially for transient solidification problems.

The LBM has been used in numerous related applications. It has been used to model turbulent flow, flow in porous media, and multi-component, multi-phase and contaminant complex flows.^23^,^24^ It can easily handle complicated geometries that change in time because of the simplified treatment of the boundaries. Of relevance to this work, the method has also been used in solidification to model dendritic growth describing heat, mass and momentum transport,^5^,^9^,^25^–^28^ the latter three using the LBM in combination with the CAM. The parallel feature of the LBM has been exploited by^1^,^3^,^28^ who have successfully simulated domains consisting of billions of elements using CPU and GPU clusters utilizing hundreds of processing units and obtaining the solution in a matter of hours.

## Numerical Method

The numerical model used in this work comprises a CAM for solidification and the LBM for hydrodynamics, linked via body forces and the solute transport equations. The fully coupled system utilizes a domain decomposition MPI based parallel framework to enable faster and larger scale calculations. This section describes the governing equation sets, discretization, coupling, parallelization and the overall algorithm.

### Cellular Automata Method

The model couples the CAM for solidification, representing crystal growth by a continuous function, with a finite difference scheme to solve for the solute diffusion. The computational cubic grid is uniform Cartesian. Three states or phases of solidification are tracked in each cell by the solid fraction parameter $\phi_{\text{s}}$: liquid, solidifying and solid. The concentration of solute in the solid and liquid at the solid–liquid interface is correlated by

1 $$ C_{\text{s}} = kC_{\text{l}} , $$

where $k$ is the partitioning coefficient of the solute. The equivalent concentration is defined as

2 $$ C_{\text{e}} = \phi_{\text{l}} C_{\text{l}} + \phi_{\text{s}} C_{\text{s}} . $$

The convective transport of the solute is governed by

3 $$ \frac{{\partial C_{\text{e}} }}{{\partial t_{\text{CAM}} }} + {\mathbf{u}} \cdot \nabla {\text{C}}_{\text{l}} = \nabla \cdot \left( {{\text{D}}_{\text{e}} \nabla {\text{C}}_{\text{l}} } \right) , $$

where ${\mathbf{u}}$ is the flow velocity in the liquid, and $D_{\text{e}} = \phi_{\text{l}} D_{\text{l}} + k\phi_{\text{s}} D_{\text{s}}$ is the equivalent diffusion coefficient. Equation 3 is discretized in an explicit form and uses a hybrid approach for the advection diffusion terms. At the interface, where ${\mathbf{u}} = 0$, the solid fraction rate of change is given by

4 $$ C_{\text{l}} \left( {1 - k} \right)\frac{{\partial \phi_{\text{s}} }}{{\partial t_{\text{CAM}} }} = - \nabla \cdot \left( {D_{\text{e}} \nabla C_{\text{l}} } \right) + \left[ {1 - \left( {1 - k} \right)\phi_{\text{s}} } \right]\frac{{\partial C_{\text{l}} }}{{\partial t_{\text{CAM}} }} . $$

The solution procedure is essentially a loop between Eqs. 3 and 4. Convective transport is calculated throughout the entire domain, and then from local changes at the interface $\phi_{\text{s}}$ is updated based on transport and partitioning by Eq. 4. Equations 3 and 4 determine the rate of solidification, but do not encapsulate any information on crystallographic orientation. This is handled by a decentered octahedral method, which essentially introduces a bias for seeding neighboring cells to begin solidifying. This bias corresponds to the underlying crystallographic orientation, such that cells along the direction of preferential growth will be seeded first. This is calculated by considering the diagonal length of a rotated octahedron growing in each cell. When this diagonal intersects a neighboring cell, the neighbor is seeded with the same underlying orientation and begins to solidify. The CAM used in this work is based on the µMatIC code, and through the parallelization and coupling process it was ensured that it gave the exact answer down to machine precision. Due to the high Lewis number and low thermal Peclet number in the cases considered, a frozen thermal field approximation is used, where the temperature is explicitly known both spatially and temporally throughout the domain. In this work the focus is on buoyancy-driven flow, which is applied in the liquid as

5 $$ {\mathbf{F}} = \rho {\mathbf{g}}\left( {1 + \beta_{\text{T}} \left( {T - T_{0} } \right) + \beta_{\text{C}} \left( {C - C_{0} } \right)} \right) $$

where $\rho$ is the density, **g** is acceleration due to gravity, $\beta_{\text{T}}$ and $\beta_{\text{C}}$ are the thermal and solutal expansion coefficients, and $T_{0}$ and $C_{0}$ are a reference temperature and concentration.

### Lattice Boltzmann Method

The LBM is formulated using non-dimensional lattice Boltzmann units, where the lattice spacing, time stepping and equilibrium density are all defined as unity. Therefore, all variables described in this section are presented in a non-dimensional form and for clarity those that have an equivalent dimensioned form in the fully coupled method are denoted with the superscript *. Scaling factors are derived from the real base SI units to scale between real and dimensionless variables, for example, ${\mathbf{u}}^{*} = {\mathbf{u}} \cdot \Delta t_{\text{LBM}} /\Delta x_{\text{LBM}}$ and the dimensionless force ${\mathbf{F}}^{\varvec{*}} = {\mathbf{F}}\Delta t_{\text{LBM}}^{2} /\rho \Delta x_{\text{LBM}}$. The LBM uses a discretized from of the Boltzmann equation that describes the evolution of a particle distribution function (PDF), $f_{i}$. The lattice Boltzmann equation is then given by

6 $$ f_{i} \left( {{\mathbf{x}}^{\varvec{*}} + {\mathbf{c}}_{i} \Delta t_{\text{LBM}}^{*} ,t^{*} + \Delta t_{\text{LBM}}^{*} } \right) - f_{i} \left( {{\mathbf{x}}^{\varvec{*}} ,t^{*} } \right) = - \frac{1}{\tau }\left( {f_{i} - f_{i}^{\text{eq}} } \right) + \Delta t_{\text{LBM}}^{*} F_{i}^{*} , $$

where the left-hand side represents the streaming processes, which govern the propagation of information to the neighboring cells. The right-hand side describes collisions or the PDF relaxation towards the local equilibrium $f_{i}^{\text{eq}}$ in time $\tau$ with an external force $F_{i}^{*}$ acting on the system. The equilibrium PDF $f_{i}^{\text{eq}}$ is defined as

7 $$ f_{i}^{\text{eq}} = \rho^{*} w_{i} \left( {1 + \frac{{{\mathbf{c}}_{\text{i}} \cdot {\mathbf{u}}^{\varvec{*}} }}{{c_{\text{s}}^{2} }} + \frac{{\left( {{\mathbf{c}}_{\text{i}} \cdot {\mathbf{u}}^{*} } \right)^{2} }}{{2c_{\text{s}}^{4} }} - \frac{{{\mathbf{u}}^{{\varvec{*}2}} }}{{2c_{\text{s}}^{2} }}} \right), $$

where $\rho^{*}$ is the fluid density, $w_{i}$ is the lattice weight coefficient, $c_{i}$ is a discrete lattice velocity, $c_{\text{s}}$ is the speed of sound, and ${\mathbf{u}}^{\varvec{*}}$ is the fluid velocity. To describe the external forces such as thermo-solutal buoyancy forces in this work, the HSD forcing scheme, named after the authors of Ref. 29 is used and given by

8 $$ F_{i}^{*} = \left( {1 - \frac{1}{2\tau }} \right)\frac{{\left( {{\mathbf{c}}_{i} - {\mathbf{u}}^{*} } \right)}}{{c_{\text{s}}^{2} }} \cdot \frac{{{\mathbf{F}}^{*} }}{m}\varvec{ }f_{i}^{\text{eq}} . $$

where $m$ is the mass. A D3Q19 lattice is used in the calculations with $c_{\text{s}}^{2} = \frac{1}{3}$, the lattice weights $w_{i}$ are given by

9 $$ w_{i} = \left\{ \begin{array}{ll} 1/3 & i = 0 \\ 1/18 & i = 1 \ldots 6 \\ 1/36 & i = 7 \ldots 18  \end{array} \right. $$

and the set of discrete lattice velocities for the model can be written as

10 $$ {\mathbf{c}}_{i} = \left\{ {\begin{array}{ll} \left( 0,0,0 \right) & i = 0 \\ {\left( { \pm 1,0,0} \right),\left( {0, \pm 1,0} \right),\left( {0,0, \pm 1} \right)} & {i = 1 \ldots 6} \\ {\left( { \pm 1, \pm 1,0} \right),\left( {0, \pm 1, \pm 1} \right),\left( { \pm 1,0, \pm 1} \right)} & {i = 7 \ldots 18} \\ \end{array} } \right. $$

Fluid properties, such as density and fluid velocities, can be calculated from the PDF by taking the velocity moments as

11 $$ \begin{array}{*{20}c} {\rho^{*} = \mathop \sum \limits_{i} f_{i} ,} & {\rho^{*} {\mathbf{u}}^{\varvec{*}} = \mathop \sum \limits_{i} f_{i} {\mathbf{c}}_{i} } \\ \end{array} + \frac{{\Delta t_{\text{LBM}}^{*} }}{2}{\mathbf{F}}^{\varvec{*}} . $$

The incompressible Navier–Stokes equations given by

12 $$ \nabla \cdot {\mathbf{u}}^{*} = 0, $$

13 $$ \frac{{\partial {\mathbf{u}}^{\varvec{*}} }}{\partial t} + {\mathbf{u}}^{\varvec{*}} \cdot \nabla {\mathbf{u}}^{\varvec{*}} = - \frac{1}{{\rho^{*} }}\nabla p + \nu^{*} \nabla^{2} {\mathbf{u}}^{\varvec{*}} + {\mathbf{F}}^{\varvec{*}} , $$

can be recovered in the low Mach number limit at small velocities by following the Chapman–Enskog multiscale analysis from (6), leading to the pressure $p$ and the kinematic viscosity $\nu^{*}$ to be expressed as

14 $$ p = \rho^{*} c_{\text{s}}^{2} , $$

15 $$ \nu^{*} = c_{\text{s}}^{2} \left( {\tau - \frac{1}{2}} \right)\Delta t_{\text{LBM}}^{*} . $$

For stability purposes, the two-relaxation-time (TRT) collision scheme is used. The TRT uses two relaxation rates. One is directly linked to the viscosity of the physical system, while the other is a free parameter that can be fine-tuned for optimal accuracy and stability.^30^–^32^

An extension of the 2D moment-based boundary method^33^–^38^ to 3D is used on the flat domain boundaries. The modified bounce-back rule, which is second order accurate,^39^ is used for interior boundaries.

### Parallelization

**Fig. 1:**
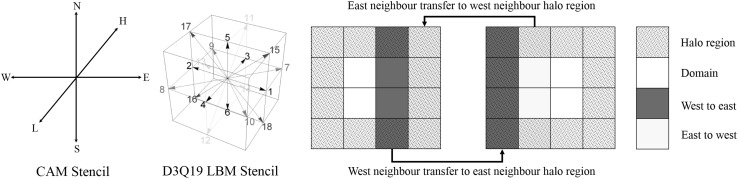
Stencils for six neighbors in CAM, the three-dimensional LBM D3Q19 and an illustration of data transfer to halo regions

Both the CAM and LBM are parallelized using domain decomposition with a boundary layer one cell wide for each process acting as a halo region to accommodate MPI-based inter-processor communication. Each process solves an equal-size sub-domain. For processes that contain domain boundaries, the boundary layer is populated with the physical boundary condition, while the halo regions contain the information from neighboring processes. The algorithm is designed so that the halo regions are updated when values from neighboring cells are required and have changed on neighboring processes. Due to the explicit nature of both the CAM and LBM, this data exchange can be reduced to one update of the halo regions per time step within each method. Updates to the physical domain boundary conditions also take place at the same time. The MPI routine begins by updating the $\hat{x}$ direction boundaries using an odd and even approach; all the odd processors send and receive values from their even east neighbor, then the even processors send and receive with their west neighbor and then the west and east halo regions are populated on all processes. The routine then does the same in $\hat{y}$ followed by $\hat{z}$. The LBM lattice stencil uses edge and corner cells, which are stored from non-orthogonal neighbors; however, using this approach additional data transfers are not required. By also passing the edge and corner values to the neighbor, after all directions have been passed, the correct information ends up in the edge and corner halo regions. Figure 1 illustrates the domain decomposition and halo region update.

### Coupling

The fully coupled CALBM model utilizes two spatial scales, one for each method, and four temporal scales, two for each method. One of the temporal scales corresponds to the time step $\Delta t_{\text{CAM}}$ for solidification and $\Delta t_{\text{LBM}}$ for flow, where $\Delta t_{\text{LBM}}$ is dimensioned. These are linked to the spatial scales through stability by the CFL condition, as both methods are fully explicit. The other temporal scales determine the strength of the coupling, such that several solidification time steps can be taken before the flow is updated, i.e., $t_{\text{CAM}} = n\Delta t_{\text{CAM }} ;\; t_{\text{LBM}} = m\Delta t_{\text{LBM}}$. For the spatial scales, each method can use different cell sizes $\Delta x_{\text{CAM}}$ and $\Delta x_{\text{LBM}}$ for solidification and flow, respectively. Selection of these scales is problem-dependent, and in this work $\Delta x_{\text{CAM}}$ is chosen to be sufficiently small to capture microstructure features, while hydrodynamic features are assumed to be larger (a reasonable assumption for low Reynolds number flow), such that $\Delta x_{\text{LBM}} \ge \Delta x_{\text{CAM}}$. For simplicity and computational efficiency, $\Delta x_{\text{LBM}}$ is chosen to be an integer multiple of $\Delta x_{\text{CAM}}$, typically 2, 3 or 4, which in three dimensions significantly reduces both the memory and CPU requirements of the LBM computation and enables simulation of large-scale domains.

**Fig. 2:**
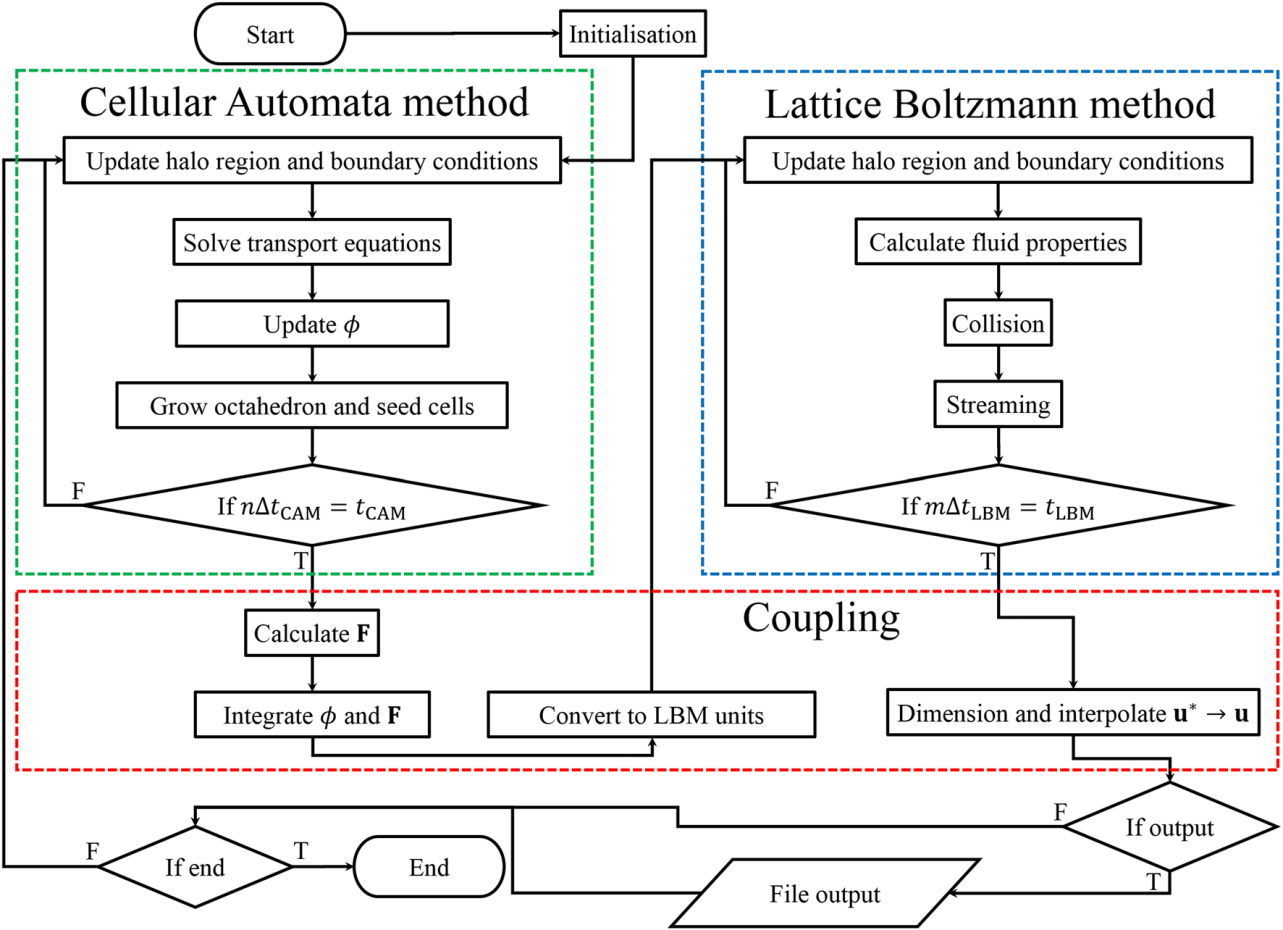
Algorithm flow chart for the fully coupled method

As shown in Fig. 2, the algorithm incorporates a two-way coupling between the CAM and the LBM. At the end of the CAM $\phi_{\text{s}}$ and ${\mathbf{F}}$ are passed to the LBM. When passing the solid fraction to the LBM, a nearest integer approach is taken; thus, if $\phi_{\text{s}} < 0.5$ the cell is assumed liquid and if $\phi_{\text{s}} \ge 0.5$ it is assumed solid. In the LBM $\phi$ is used to update the internal no-slip velocity boundary conditions at the liquid–solid interface, while ${\mathbf{F}}$ provides an external force used in the LBM. Between successive calls to the CAM ${\mathbf{F}}$ may change, for example, buoyancy forces from density variation through transport of solute. By using the previous known velocity and updated values for $\phi$ and ${\mathbf{F}}$, the LBM calculates new values for ${\mathbf{u}}$ over the time interval $t_{\text{LBM}}$. The new values of ${\mathbf{u}}$ are then passed back to the transport equation, linking both the CAM and the LBM.

Due to the disparity in spatial scales and dimensionality of the two methods, remedial steps are required between each method. When passing from the CAM to the LBM, $\phi$ and ${\mathbf{F}}$ are integrated over $\Delta x_{\text{LBM}}^{3}$ and ${\mathbf{F}}$ is converted into non-dimensional LBM units. Conversely, when passing back from the LBM to the CAM, ${\mathbf{u}}$ is interpolated on to the solidification mesh and dimensioned. By taking multiple time steps within each method, the frequency in which the remedial steps are taken reduces, but at the cost of coupling strength. While the remedial steps are not computationally demanding, a notable performance drop would occur when $t_{\text{CAM}} = \Delta t_{\text{CAM}} = t_{\text{LBM}} = \Delta t_{\text{LBM}} = {\text{min }}\left( {t_{\text{CAM}} ,\Delta t_{\text{CAM}} ,t_{\text{LBM}} ,\Delta t_{\text{LBM}} } \right)$. The required coupling strength is problem dependent. As an example, it can be estimated by considering the typical interface velocity as 10 $\mu {\text{m/s}}$ with $\Delta x_{\text{CAM}} = 10\,\mu {\text{m}}$ and $\Delta t_{\text{CAM}} = 1\;{\text{ms}}$ gives 1000 $\Delta t_{\text{CAM}}$ steps to solidify a single cell. Alternatively, by considering the time a fluid packet would take to cross a cell, with maximum velocities of *O*(1 mm/s) give a characteristic time scale of 10 $\Delta t_{\text{CAM}}$.

Although selection of cell size and time stepping is problem dependent, there are also numerical constraints in terms of stability and convergence. The advection-diffusion equation for solute transport is handled by an explicit hybrid finite difference method. The CFL condition for this scheme is $\frac{{2D\Delta t_{\text{CAM}} }}{{\Delta x_{\text{CAM}}^{2} }} < 1$. In the cases presented, typical values of $\Delta x_{\text{CAM}} = 10 \;\mu {\text{m}}$ and $\Delta t_{\text{CAM}} = 2$ ms are used giving CFL = 0.08. The LBM is also fully explicit with the CFL condition $\frac{{|u^{*} |\Delta t^{*} }}{{\Delta x^{*} }} < 1$, which is automatically satisfied because of the small velocity constraint $u^{*} < c_{\text{s}} \approx 0.577$. The $\Delta {{t}}_{\text{LBM}}$ in the LBM is calculated using the viscosities as $\Delta {{t}}_{\text{LBM}} = \frac{{\nu^{*} }}{\nu }\Delta x_{\text{LBM}}^{2}$.

## Experimental Setup

**Fig. 3:**
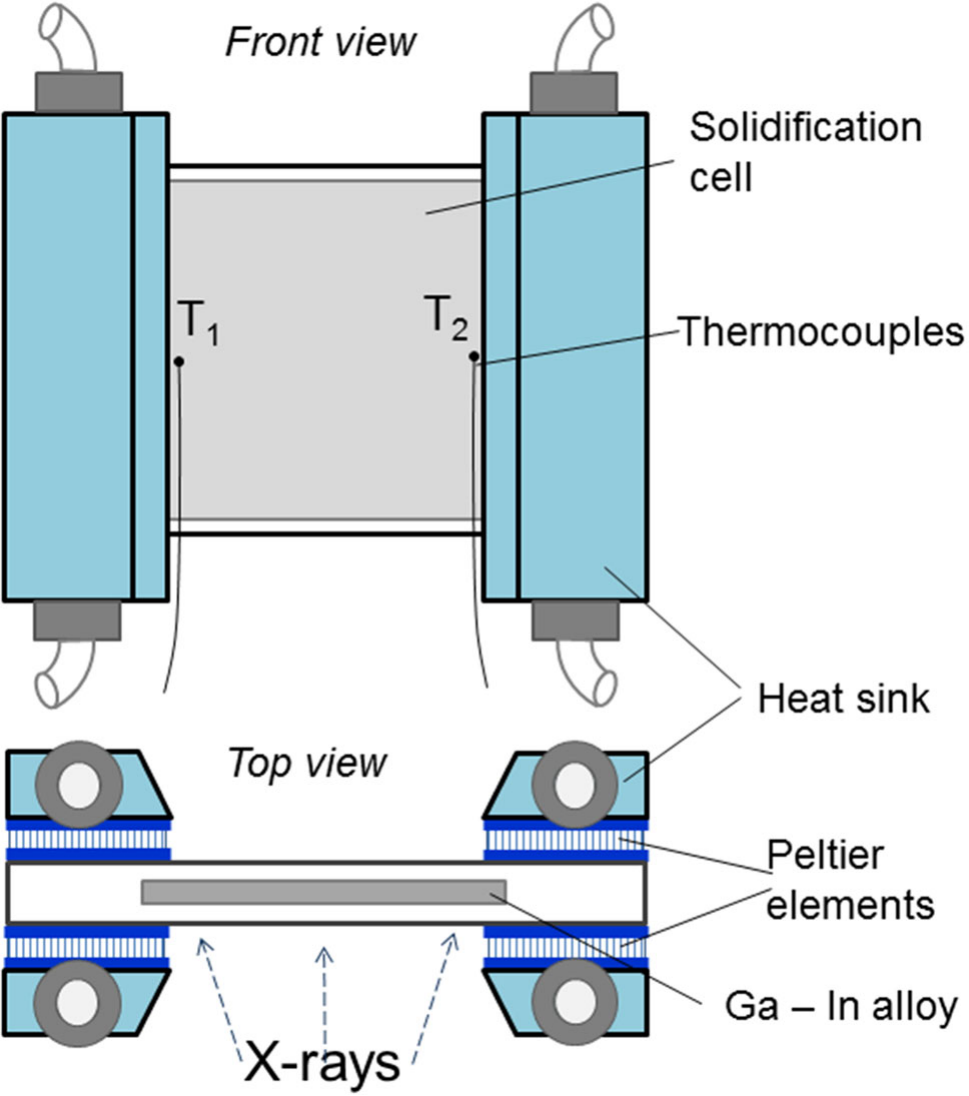
Schematic view of the solidification cell

The results section looks at four test cases; the third and fourth cases compare the numerical model to experiments for solidification of a differentially heated cavity. For this case, the solidification experiments are conducted in a Hele-Shaw cell (30 × 30 × 4 mm^3^) made of quartz with a liquid metal volume of 29 × 29 × 0.15 mm^3^ (see Fig. 3). The cell is filled with the low-melting-point hypereutectic Ga-25wt.%In alloy prepared from gallium and indium of 99.99% purity. Two pairs of Peltier elements are mounted as a heater and as a cooler on the right and left walls of the solidification cell, respectively. The distance between the heater and the cooler is 25 mm. The synchronized regulation of the power of both Peltier elements by means of a PID controller unit allowed the cooling rate and temperature gradient to be adjusted during the process. The temperature difference Δ*T* between the heater and cooler is measured using two miniature K-thermocouples, which are contacted to the outer surface of the cell near the edge of Peltier elements as shown in Fig. 3. The distance between thermocouples *T*_1_ and *T*_2_ is 23 mm. The accuracy of the temperature control is ± 0.3 K. In the present experiments, a cooling rate of 0.01 K/s and a temperature gradient of 2.5 K/mm were applied. The solidification setup is mounted on a three-axis translation stage between a microfocus x-ray source (XS225D-OEM, Phoenix x-ray, Germany) and an x-ray detector (TH9438HX 9″, Thales, France). The rectangular observation window is about 25 × 29 mm^2^. In situ and real-time observation of the solidification process is realized with an acquisition rate of 1 frame per 1 s and an effective pixel size of about 45 µm at the CCD sensor. This setup is similar to experiments conducted with a vertical thermal gradient, where further details can be found in Refs. 40 and 41.

The x-ray radiography delivers a two-dimensional projection of the local density in the slit container corresponding to the distribution of the relative brightness in the acquired images. The calibration for composition measurements is performed by using area reference measurements of cells filled with pure Ga liquid and Ga-25wt.%In. The analysis of the solidification front velocity and the image processing description can be found in Refs. 41 and 42. Table I provides some essential material properties used for the numerical modeling. These material data are also used in Case 3.

**Table I:** Material property values used for simulation

Property	Variable	Value	Unit
Density Ga	$\rho_{\text{Ga}}$	6095	kg m^−3^
Density In	$\rho_{\text{In}}$	7020	kg m^−3^
Kinematic viscosity	$\nu$	3.28 × 10^−7^	m^2^ s^−1^
Partitioning coefficient	$k$	0.5	–
Solute diffusivity	$D_{l}$	2 × 10^−9^	m^2^ s^−1^
Liquidus slope	$m_{\text{l}}$	2.9	K wt.%^−1^
Solutal expansion coefficient	$\beta_{C}$	1.66 × 10^−3^	wt.%^−1^
Thermal expansion coefficient	$\beta_{\text{T}}$	1.18 × 10^−4^	K^−1^

## Results

This section shows four test cases: the first two, which verify individual modules of the coupled system, and the final two present validations against the GaIn experiments described in “Experimental Setup” section. The first is a classic and relevant benchmark case demonstrating the accuracy of the LBM in vortex shedding producing a von Kármán vortex street. The second case is a benchmark test of the CAM with and without forced convection. The third and fourth cases are applications of the coupled method in the Ga-25wt.%In system capturing meso-/macroscale features from a microstructure perspective. Case 3 investigates directional solidification with a vertical thermal gradient and the formation of plumes of solute, which are related to freckle defect formation. Case 4 looks at solidification of a laterally differentially heated cavity and the interaction between solutal and thermal buoyancy forces. Both cases are compared with experiments. In all cases material properties are assumed to be temperature and composition invariant per phase and the flow is assumed to be laminar and incompressible.

### Case 1: LBM Benchmark Case—von Kármán Vortex Street

Flow past a cylinder is one of the oldest hydrodynamic benchmark cases and has been investigated for many decades. As flow passes the cylinder, it detaches and vortices are shed downstream. It was chosen as it is a fully transient system that reaches a periodic solution. One way to compare results is by relating the Reynolds number, $Re = \frac{uL}{\nu }$, and the Strouhal number, $St = \frac{L\omega }{{u_{\infty } }}$, where $\omega$ is the shedding frequency and $u_{\infty }$ is the free stream velocity. The grid size for this 2D transient benchmark case is $L \times H = 1200 \times 600$. Numerical results are obtained at five different Re ranging from 50 to 250, which is the laminar regime of the flow. The grid size and the free stream velocity are fixed while the dimensionless viscosity varies for different Re. Free stream velocity, $u_{\infty } = 0.1$, is applied to the west, north and south boundaries, and a pressure outlet is used at the east boundary. A cylinder of diameter $D = H/15$ is placed at $\left( {H/2, H/2} \right)$ with the wake zone of length $22D$.

**Fig. 4:**
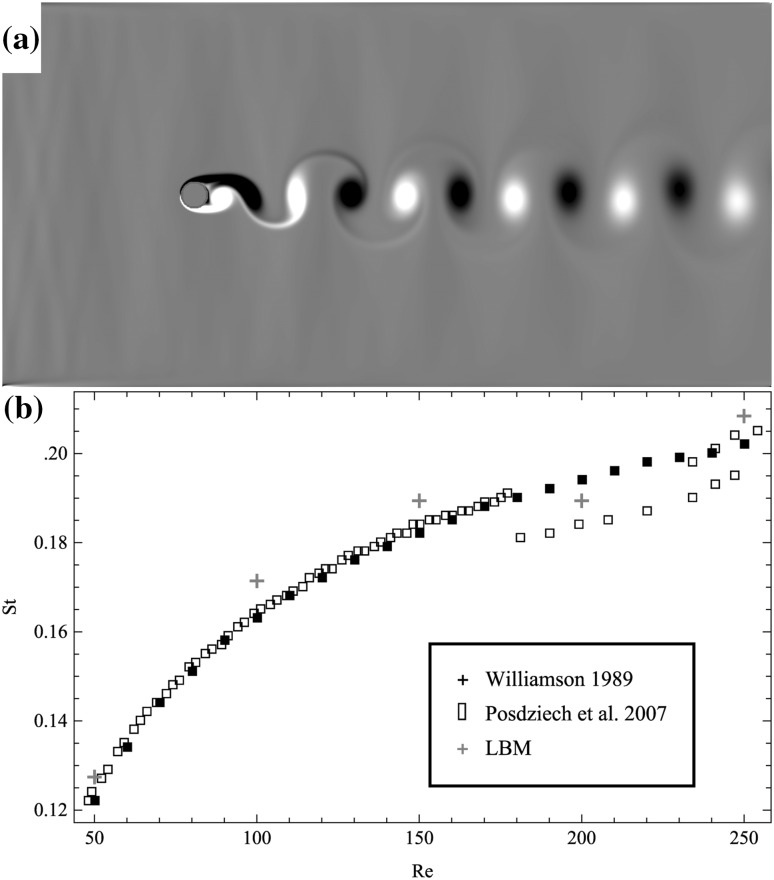
von Kármán vortex street. (a) Simulation results of vortex shedding. (b) Comparison to literature.^43^,^44^

Figure 4a shows the von Kármán vortex street results produced by the LBM and Fig. 4b shows a comparison for several different Re flows. The results demonstrate the LBM can accurately reproduce the behavior of this benchmark case.

### Case 2: CAM/LBM Benchmark Case—Solidification in Forced Convection

**Fig. 5:**
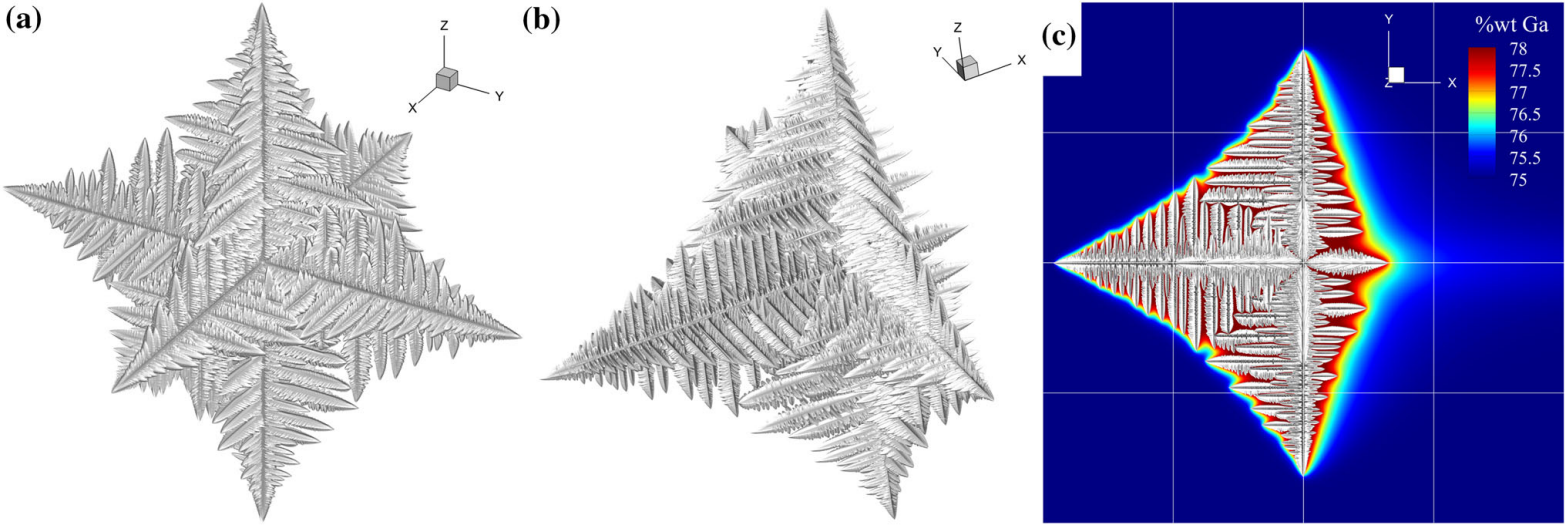
Free equiaxed growth. (a) No flow. (b, c) Forced convection of ${\mathbf{u}} = 100 \;\upmu{\text{m}}/{\text{s}}$ in $+ \hat{x}$ viewed at different angles

Consider a low, 10-K, undercooled melt with a single homogeneous nucleus at the center of the domain. In this idealized case, thermophysical properties for Ga-25wt.%In are used, but gravitational forces are neglected. Under stagnant flow conditions solidification will progress as a symmetric equiaxed dendrite, the morphology of which is shown in Fig. 5a. With no flow, the LBM code is not solved. When an incident flow of 100 µm/s is applied, the dendrite exhibits a bias in growth direction (Fig. 5b), with the incident tip increasing in growth velocity, the tangential tips remaining relatively unchanged and the downstream tip significantly retarded. This general behavior is in agreement with results from the literature Refs. 5 and 45–50. The forced convection transports bulk undercooled fluid towards the incident tip, promoting growth, and then transports the high-solute concentration downstream, where growth is stunted, as shown in Fig. 5c. As the dendrite evolves, the solidified regions act as an internal zero velocity boundary condition and the fluid flow goes around the dendrite. This test case was run using 1 billion computational (1024^3^ cells) 10-µm computational cells for 100-k time steps (200 s) and highlights the full coupling between the LBM and the CAM codes. The boundary conditions for flow are a fixed velocity on the west face, fixed pressure boundary for the east face and zero Neumann conditions for the remaining faces.

### Case 3: Plume Formation in Ga-25wt.%In Solidification

Case 3 focuses on a particular phenomenon encountered in alloys that are subject to strong solutal buoyancy effects during solidification. The ejected component is lighter than the bulk liquid melt, causing plumes of solute to spring from the solidification interface. Such alloys are widely encountered in industry, notably Ni-based superalloys in the production of turbine blades. Ga-25wt.%In exhibits similar behavior, but as a low temperature alloy is used as a model system. Thin-sample radiography experiments of directional solidification of Ga-25wt.%In have been conducted capturing the plumes of high-concentration gallium Refs. 40 and 51–53. The thermal gradient is in the same direction as gravity.

The numerical model represents a 16.8 mm × 200 µm × 16.8 mm domain size, with 10 µm cell sizes. Initially, 80 equally spaced nuclei with random crystallographic orientations are placed on the lower face of the domain. The west and east faces are periodic, the south, north and low faces are zero velocity boundaries, and the high face is a fixed pressure boundary. Although the experiments are for a thin sample with a large aspect ratio, it is necessary to capture the thin third dimension, including the effect of the walls. The buoyant plumes and developing stable channel formations are fed by interdendritic flow and flow between the sample wall and dendrites,^21^ which if modeled in two dimensions, cannot exist.

**Fig. 6:**
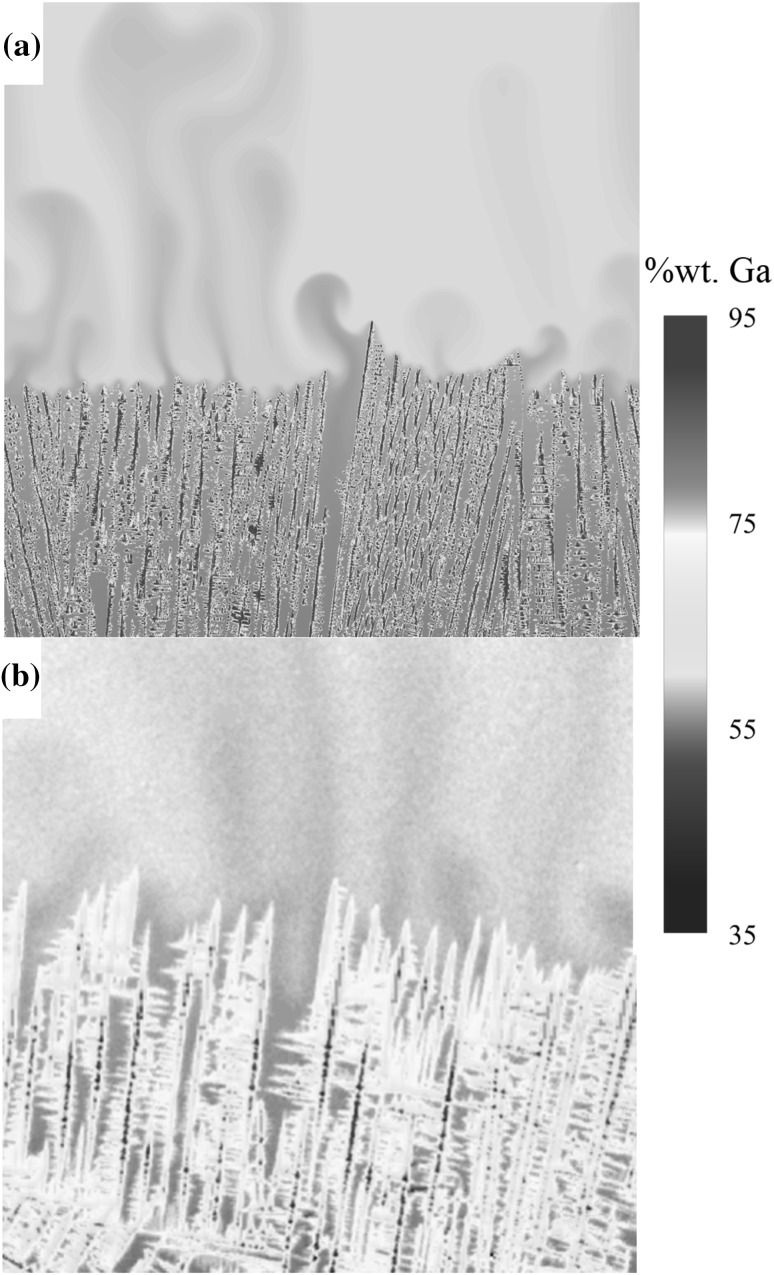
Plume formation results in Ga-25wt.%In alloy. (a) Numerical. (b) Experimental. Reprinted from Ref. 53

Figure 6 shows a comparison between numerical results generated from the CALBM and experimental results.^53^ This result shows that with the capability to model large domain sizes, it is possible to capture both the microscale effects of dendritic growth and interdendritic flow with also the mesoscale effects of interacting plumes and interacting grains in a single simulation from a microstructure perspective. The numerical result is taken after 1.8 million time steps (3600 s physical time). The domain tracks the interface such that information is lost at the low face as solidification progresses and far field conditions are applied at the top face. The simulation took 114 h on 120 cores.

### Case 4: Ga-25wt.%In Solidification Subject to a Horizontal Thermal Gradient

**Fig. 7:**
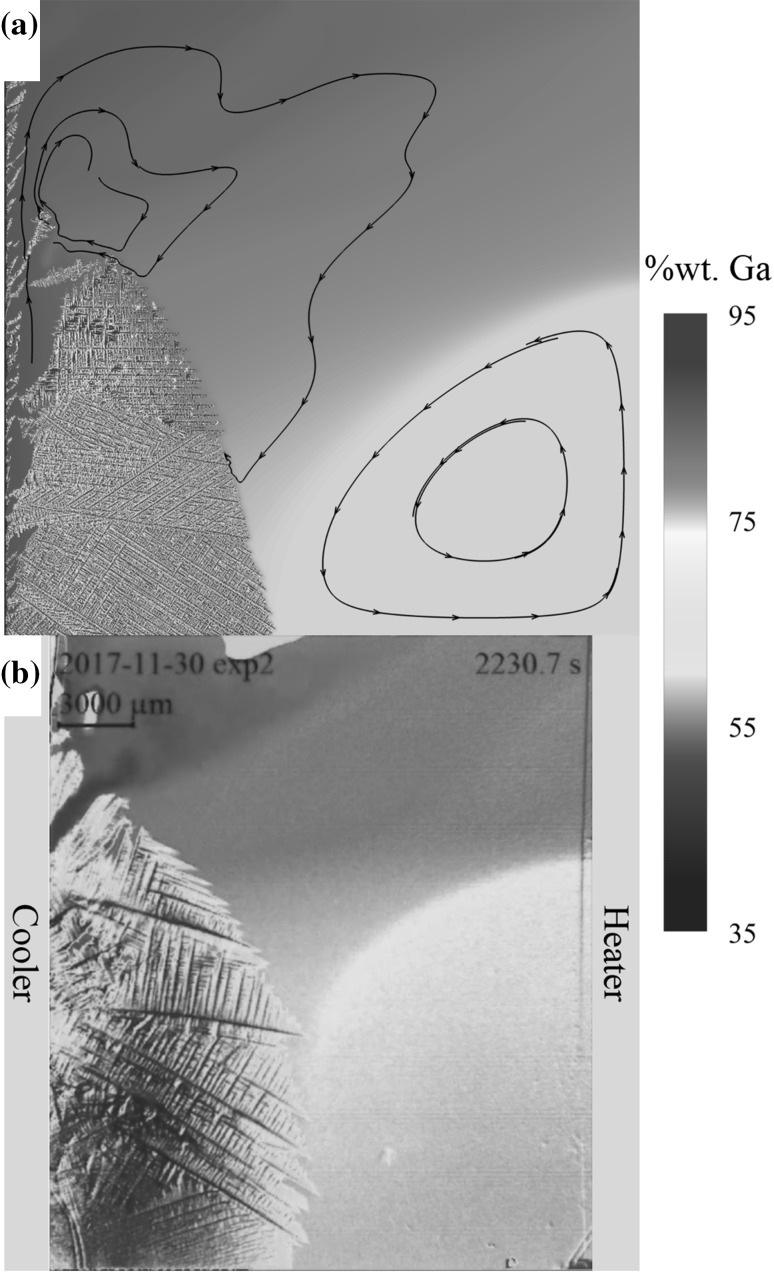
Solidification with a horizontal thermal gradient. (a) Numerical. (b) Experimental

In this case, the domain is split into $3072 \times 16 \times 3072 = 151$ million cells with $\Delta x = 9.375 \;\mu {\text{m}}$. This corresponds to a domain size of $28.8\;{\text{mm}} \times 150\;\mu {\text{m}} \times 28.8\;{\text{mm}}$, closely representing the full solidification experiment. In this case all boundaries are zero velocity representing the sample walls. The domain is decomposed over $16 \times 1 \times 8 = 128$ processors. Physical parameters including the thermal gradient and cooling rate are calibrated based on experimental observations. Thirty-two nuclei with random crystallographic orientations are equally distributed on the cold west wall of the domain. Figure 7 shows a comparison between the numerical model and the experimental results. The numerical result is taken at 2400 s (1.2 million time steps) taking 72 h to compute, while the experiment is taken at 2230 s after the first dendrite is observed. The slight disparity in time can be attributed to relatively large uncertainties in initial conditions. It is not possible to observe the nucleation events as they are covered by the cooler, and it is uncertain if there is any initial undercooling at nucleation. The results are in qualitatively good agreement for both the interface shape and solute concentration profile.

With an initial homogeneous composition, fluid flow is dominated by thermal buoyancy forces generating a counterclockwise rotating vortex with a direct analogy to a differentially heated cavity. However, as the system cools and solidification progresses, solute is ejected with increasing concentration. The strong but localized solute buoyancy forces overcome the thermal buoyancy force and a secondary vortex forms in the vicinity of the solidification front. Solute is transported to the top of the sample, stunting growth in this region. As more solute is transported to the top surface of the sample, Ga-rich liquid extends across the top surface, forming a competing vortex and constraining the thermally driven vortex. As solidification progresses further, a stable solute-rich channel forms, fed by interdendritic flow lower down the sample. This stable channel continues to feed the growing solute-driven vortex. The solutal buoyancy-driven vortex transports high-concentration Ga to the boundary of the two vortices, while the thermally driven one drives the bulk concentration to the boundary. This leads to a stratification of concentration, which is clearly visible in the experimental results. The overall mechanism is captured by the numerical modeling; the location of the stable channel and the competing the vortices both compare favorably with the experimental observations. This result demonstrates that the coupled CALBM has the capability to capture meso-macroscale effects from a microstructural perspective; in this case the entire experiment is modeled. This allows for direct modeling of constraints, for example, the sample end walls, where in many studies only sections of experiments can be captured and approximate boundary conditions such as periodic or open boundaries are necessary, but may not be representative.

## Performance

In this section a summary of the performance of the various cases is given. In the cases presented the domain sizes vary from *O*(200 million to 1 billion) cells. The parallel efficiency of the CALBM was found to vary between 60% and 70%, as additional computations are required for the inter-processor communication while updating the halo regions. The computational requirement of the solvers scales with the cube of the domain length, while the communication scales as the square of the domain length. Consequently, the higher efficiency corresponds to the larger domain sizes. However, with increasing domain size per processor, the ratio of run time to simulated time increases. For example, case 4 took around 3 days to simulate 2400 s of physical time (1.2 million time steps). However, on a single processor this would take an unfeasible amount of time, around 270 days.

Case 2 provides a comparison of CAM and LBM. For free growth without flow the simulation took 14 h to calculate 100-k CAM time steps, while with flow it took 19.5 h for the same number of steps. Approximately 2/3 of the simulation was resolving solidification; however, as $\Delta x_{\text{LBM}} = 4\Delta x_{\text{CAM}}$ the number of LBM cells was 64 times smaller. On a one-to-one scale, resolving the hydrodynamics would take over 32 times longer than solidification, highlighting the necessity for variable length scales between solidification and hydrodynamics. However, as LBM has been shown to scale very well with GPUs, such approximations may be mitigated in the future.

## Conclusion

For simulating large-scale domains on a microstructure scale that encompass small components or entire experimental setups, the coupled CALBM code has been shown to provide accurate results at both the micro- and mesoscales. Each of the modules of the CALBM was validated against classic benchmark test cases. The LBM was shown to accurately predict the well-known relationship between Re and St for low Re flow past a cylinder. The microstructure modeling was verified by simulation of a single free-growing equiaxed dendrite in a low-undercooled melt. Adding incident forced convection onto one of the dendrite arms, the CALBM was shown to give similar preferential growth to results in the literature. The CALBM was then applied to two large-scale problems with both micro- and mesoscale features using the Ga-25wt.%In alloy. The first showed directional solidification with a vertical thermal gradient, where the generation of solute plumes due to solutal buoyancy led to the formation of solute channels in the microstructure. The second investigated solidification of a differentially heated cavity with a horizontal thermal gradient, where a competition between thermal and solutal buoyancy forces led to two large-scale counter-rotating vortices. Ejected solute is fed by the large solutal buoyancy force driving flow in the interdendritic region. In both of the validation cases, favorable agreement at both the micro- and mesoscale was found between the numerical and experimental results.

## Future Work

The CAM and LBM were chosen for this work as they represent potentially the largest microstructure-length-scale computational tool and fastest transient flow simulator respectively. They also lend themselves to massive parallelization. In the examples presented, parallelization was only conducted on a CPU over MPI basis. These methods, certainly LBM, can see huge speed increases when utilizing GPUs. However, there will be an increase in communication overheads from transferring data between the CPU and GPU of field data and to keep the halo regions updated via MPI. Such GPU implementations have been realized in other related methods with a high degree of success, and as such they are worth pursuing for the CALBM. With the ability to simulate *O*(1 billion) cells in a timely manner, the entire microstructure of small components *O*(100 mm × 100 mm × 100 mm) could be readily predicted. The results presented here provide a qualitative agreement with the experimental results; however, with increasing cell size there will be a loss of accuracy in capturing microstructural features, but this will allow for even larger domains. A future study is planned to quantify the behavior of this error. This will encompass a direct comparison of solute concentration profiles between the numerical and experimental results.
